# Associations of Non-Alcoholic Beverages with Major Depressive Disorder History and Depressive Symptoms Clusters in a Sample of Overweight Adults

**DOI:** 10.3390/nu12103202

**Published:** 2020-10-20

**Authors:** M. Ángeles Pérez-Ara, Margalida Gili, Marjolein Visser, Brenda W.J.H. Penninx, Ingeborg A. Brouwer, Ed Watkins, Matt Owens, Mauro García-Toro, Ulrich Hegerl, Elisabeth Kohls, Mariska Bot, Miquel Roca

**Affiliations:** 1 Institut Universitari d’Investigació en Ciències de la Salut, Idisba, Rediapp, University of Balearic Islands, 07122 Palma de Mallorca, Spain; m.perez@uib.es (M.Á.P.-A.); mgili@uib.es (M.G.); mauro.garcia@uib.es (M.G.-T.); 2 Department of Health Sciences, Faculty of Science and the Amsterdam Public Health Research Institute, Vrije Universiteit Amsterdam, 1081 HV Amsterdam, The Netherlands; m.visser@vu.nl (M.V.); ingeborg.brouwer@vu.nl (I.A.B.); 3 Amsterdam UMC, Department of Psychiatry, Amsterdam Public Health Research Institute, Vrije Universiteit Amsterdam, de Boelelaan 1117, 1081 HV Amsterdam, The Netherlands; b.penninx@amsterdamumc.nl (B.W.J.H.P.); m.bot@ggzingeest.nl (M.B.); 4 Department of Psychology, University of Exeter, Exeter EX4 4QG, UK; E.R.Watkins@exeter.ac.uk (E.W.); M.Owens-Solari@exeter.ac.uk (M.O.); 5 Department of Psychiatry, Psychosomatics, and Psychotherapy, Goethe-Universität Frankfurt, 60438 Frankfurt a.M., Germany; ulrich.hegerl@deutsche-depressionshilfe.de; 6 Department of Psychiatry and Psychotherapy, Medical Faculty, University Leipzig, 04109 Leipzig, Germany; Elisabeth.Kohls@medizin.uni-leipzig.de

**Keywords:** depression, depressive symptoms clusters, soft drinks, coffee, tea

## Abstract

Background: Meta-analysis of observational studies concluded that soft drinks may increase the risk of depression, while high consumption of coffee and tea may reduce the risk. Objectives were to explore the associations between the consumption of soft drinks, coffee or tea and: (1) a history of major depressive disorder (MDD) and (2) the severity of depressive symptoms clusters (mood, cognitive and somatic/vegetative symptoms). Methods: Cross-sectional and longitudinal analysis based on baseline and 12-month-follow-up data collected from four countries participating in the European MooDFOOD prevention trial. In total, 941 overweight adults with subsyndromal depressive symptoms aged 18 to 75 years were analyzed. History of MDD, depressive symptoms and beverages intake were assessed. Results: Sugar-sweetened soft drinks were positively related to MDD history rates whereas soft drinks with non-nutritive sweeteners were inversely related for the high vs. low categories of intake. Longitudinal analysis showed no significant associations between beverages and mood, cognitive and somatic/vegetative clusters. Conclusion: Our findings point toward a relationship between soft drinks and past MDD diagnoses depending on how they are sweetened while we found no association with coffee and tea. No significant effects were found between any studied beverages and the depressive symptoms clusters in a sample of overweight adults.

## 1. Introduction

Depression is an important public health issue [1,2]. According to the World Health Organization [3], depression is a common mental disorder worldwide with more than 300 million people affected. The disorder is influenced by social, environmental, psychological, behavioral, genetic, hormonal, immunological, biochemical and also neurodegenerative factors [4,5,6]. Even though some of these factors are immutable, variables related to behavioral factors such as lifestyle are modifiable.

Emerging evidence suggests that diet and nutrition have a significant impact on depressive symptoms [7,8]. Soft drinks, along with coffee or tea are the most consumed non-alcoholic beverages [9]. A frequent consumption of soft drinks and/or carbonated beverages sweetened with sugar or with non-nutritive sweeteners (NNSs), has been associated with health problems, with the risk being greater when the sweetener is sugar [10,11,12]. Soft drink consumption either sweetened with sugar or with NNSs, was also associated with depressive symptoms and suicidal thoughts in cross-sectional studies [13,14,15,16] and in a prospective study with a cohort of older USA adults [17].

In relation to the underlying mechanisms, a growing body of research shows that added sugars or sugar-sweetened beverages and/or food may play an important role in the risk of depression [18,19,20]. Sanchez-Villegas et al. [21] found that added sugars but not sugar-sweetened beverages were associated with depression.

Coffee is one of the most consumed drinks in the world after water [22], comprising 80% of the drinks that contain caffeine [23]. Coffee has good nutritional properties. Its lifelong consumption produces positive effects on health. For example, inverse correlation was found between its consumption and the risk of developing different diseases [22,24,25], and even the risk of mortality [26]. Several meta-analyses of observational studies [27,28,29] indicate that coffee drinking is inversely associated with depression symptomatology. The mechanisms of the inverse association of coffee in the literature might be mediated by the presence of bioactive constituents, such as caffeine, chlorogenic acid, ferulic acid and caffeic acid. These constituents have biological properties that alter the parameters associated with the neuroinflammatory hypotheses of depression including anti-inflammatory and antioxidant properties [30]. Specially, caffeine mimics the dopamine receptor agonists and inhibits both adenosine A1 and A2A receptors. Thus, it reveals a potential antidepressant activity [31]. Regarding caffeinated soft drinks such as energy drinks, most studies of chronic use provided evidence to suggest that energy drinks are associated with mental health problems [32].

Tea consumption has also been attributed to having multiple beneficial health properties [33]. The meta-analyses of observational studies by Farajzadeh et al. [34] and Dong et al. [35] found a significant association between tea consumption and depression symptomatology (35 to 37% lower risk of depression among participants who consumed tea), whereas Grosso et al. [28] concluded that although tea seems to protect against depression, results are not conclusive and should be taken with caution. L-theanine, polyphenols and polyphenol metabolites constituents included in most types of tea are able of functioning through multiple pathways simultaneously to reduce the risk of depression [36].

Previous studies based on the fifth Korean National Health and Nutrition Examination Survey (KNHANES) examined the associations between the frequency of consumption of coffee and/or tea and self-reported lifetime depression in a general Korean population [37,38]. Both studies concluded that frequent consumption of coffee was inversely associated with self-reported lifetime depression prevalence. Furthermore, Kim and Kim [37] also found the same association between frequent green tea consumption and depression prevalence. The main limitations of these studies were the self-reporting of depression (participants had not been diagnosed by a mental health professional), and the authors did not include an adjustment for overall diet score in the analysis.

In the most recent meta-analysis of observational studies carried out to date [27], the authors concluded that soft drinks may increase the risk of depression, while high consumption of coffee and tea may reduce the risk of depression. A better understanding of the relationship between the non-alcoholic beverages and mental health would be necessary. Depression it is often a condition with relapses and recurrences [39]. Providing knowledge about the relationship between previous depressive episodes and beverages consumption could help to prevent future depressive episodes by promoting healthy drinking patterns. In addition, depressive symptom patterns/clusters have not yet been studied. Previous works proposed to differentiate three symptom clusters [40,41]: mood symptoms (e.g., feeling sad, anxious, reduced pleasure or interest), cognitive symptoms (e.g., concentration difficulties, pessimism, suicidal thoughts), and somatic/vegetative symptoms (e.g., sleeping problems, appetite increase/decrease, aches and pains).

In the MooDFOOD [42,43] prevention trial, a multicenter 2 × 2 factorial randomized clinical trial to study the effect of multinutrient supplementation and food-related behavioral activation therapy (FBC) on the prevention of major depressive disorder (MDD) among 1025 overweight adults with subsyndromal depressive symptoms was conducted; results showed that multinutrient supplementation compared with placebo and food-related behavioral activation therapy compared with no-therapy did not reduce episodes of MDD at a 12-month follow-up. In the context of this large-scale prevention trial, the aims of the present work were to examine by exploratory post-hoc analysis: (1) the relationship between certain non-alcoholic beverages (soft drinks, coffee and tea) and past MDD diagnosis; and (2) to explore the potential differences in depressive symptom clusters (mood symptoms, cognitive symptoms, and somatic/vegetative symptoms) in relation to these non-alcoholic beverages consumption.

## 2. Materials and Methods

### 2.1. Design

Baseline and 12-month-follow-up data were collected from four countries participating in the MooDFOOD prevention trial. MooDFOOD is a multi-country collaborative European project on the role of diet, food-related behavior, and obesity in the prevention of depression, registered as a clinical trial (www.clinicaltrials.gov; number of identification: NCT02529423). The main objective of MooDFOOD was to examine the feasibility and effectiveness of two different nutritional strategies to prevent a new episode of MDD in high-risk overweight people with subsyndromal symptoms of depression. The trial design was a 2 × 2 factorial randomized clinical trial. Participants were allocated one of four intervention groups: (1) daily multi-nutrient supplements (1412-mg omega-3 fatty acids, 30-μg selenium, 400-μg folic acid, and 20-μg vitamin D3 plus 100-mg calcium); (2) placebo; (3) daily multi-nutrient supplements plus food-related behavioral activation therapy (specific nutritional advice on improving food-related behaviors and to follow a Mediterranean style diet); (4) placebo plus food-related behavioral activation therapy.

A detailed description of the MooDFOOD study design and protocol can be found in Roca et al. [43].

### 2.2. Study Population

A total of 1025 participants from four different European countries (The Netherlands, United Kingdom, Germany and Spain) were recruited between September 2015 and October 2016. The recruitment consisted of MooDFOOD brochures and posters in public areas, websites, local advertisements on social media, and articles in local newspapers, mailings to registered subjects in the general practice settings or in other registers (e.g., city registers), invitation letters, press releases to the national press as well as recruitment via other studies and trials conducted at the four sites.

Inclusion criteria were: aged between 18 and 75 years old; meeting criteria for being overweight or obese according to a body mass index (BMI) between 25 and 40 kg/m^2^; and report subsyndromal symptoms of depression measured by the Patient Health Questionnaire (PHQ-9) score of at least 5 points [44]. Exclusion criteria were: current episode of MDD according to the psychiatric Diagnostic and Statistical Manual of Mental Disorders, Fourth Edition (DSM-IV) [45] criteria (including the past 6 months) assessed by a structured MINI International Neuropsychiatric Interview 5.0 (MINI 5.0; [46]; current eating disorder; history of psychosis, bipolar disorder, substance dependence or other severe psychiatric disorder that requires specialized clinical attention; history of bariatric surgery; currently pregnant or breastfeeding; current severe life-threatening physical disease (assessed using self-report); severe cognitive impairment limiting the participation in the study assessed by the ability to complete the screening instruments in an adequate manner; currently receiving a behavioral intervention that interferes with MooDFOOD prevention trial intervention; and finally, unwilling to stop using specific dietary supplements that are used or are competing with MooDFOOD prevention trial.

All subjects gave their informed consent for inclusion before they participated in the study. The study was conducted in accordance with the Declaration of Helsinki and it was approved by the Ethics Committee of the Govern de les Illes Balears, Palma, Spain (10 March 2015, number IB 2515/15 PI); the Ethics Committee of the University Leipzig, Medical Faculty, Germany (30 June 2015, number 167/15-ff); the Ethics Committee of the VU Medical Center Amsterdam, the Netherlands (8 July 2015, number 2015.160); and the NHS National Research Ethics Service (NRES) Committee for University of Exeter, SouthWest, UK (3 August 2015, number 15/SW/0153). Eligible participants underwent a face-to-face baseline interview by a trained research assessor. Baseline interviews included the assessment of a lifetime history of depression through MINI 5.0 depression section [46], sociodemographic status, physical measurement (height, weight, waist circumference, blood pressure, blood sampling) and self-administered questionnaires. They were followed up for 12 months. Participants were allocated to one of four intervention groups: (1) daily multi-nutrient (2) placebo; (3) daily multi-nutrient supplements plus food-related behavioral activation therapy; (4) placebo plus food-related behavioral activation therapy.

### 2.3. Participants

A total of 941 participants who fulfilled the Food Frequency Questionnaire at baseline and at a 12-month follow-up were included in the present work. Participants with no or incomplete data on the on the food intake self-report questionnaire were excluded (*n* = 84).

### 2.4. Measures

#### 2.4.1. Presence of Lifetime MDD

MINI International Neuropsychiatric Interview 5.0 (MINI 5.0; [46]). A brief standardized diagnostic interview used to assess disorders described in the Diagnostic and Statistical Manual of Mental Disorders, Fourth Edition (DSM-IV; [45]). The Depression section of the interview was specifically used to assess lifetime history of MDD.

#### 2.4.2. Depression Measurements

Inventory of Depressive Symptomatology-Self Report [47]. A 30-item self-administered questionnaire ranging from 0 to 84 was used to assess the severity of depressive symptoms in the last week. Items are rated on a scale from 0 (no symptom present) to 3 (severe impairment). A cut-off point of 18 or above indicate the presence of clinically relevant depressive symptomatology. The scale presents acceptable psychometric properties [48,49]. In accordance with previous studies [39,40], individual symptoms were categorized into symptoms clusters: mood cluster (16 item ranged from 0 to 30); cognitive cluster (4 items ranged from 0 to 12); and somatic/vegetative cluster (ranged from 0 to 48). See Supplementary Table S1.

#### 2.4.3. Non-Alcoholic Beverages Consumption

Food Frequency Questionnaire (GA2LEN-FFQ; [50]). This is a self-report questionnaire to assess dietary intake in the last 12 months. It includes a wide range of foods and nutrients (a list of 200 foods and drinks) representative of European diet. Foods are classified into groups by using the European Food Group (EFG) classification system [51]. The Beverages section was used in the present study, specifically non-alcoholic beverages (carbonated/soft drinks with sugar or with NNSs measured in units of 200 mL) and tea/coffee groups measured in units of a cup. Whether sugar was added to tea or coffee was not recorded. The frequency of consumption is assessed in eight categories: never/rarely, 1–3 times a month, once a week, 2–4 times a week, 5–6 times a week, once a day, 2–3 times a day, 4 or more times a day.

#### 2.4.4. Lifestyle and Health Status

Adherence to the MooDFOOD diet was assessed in a range from 0 to 77 based on the GA2LEN-FFQ [50]. Higher scores indicated a better adherence to the MooDFOOD diet based on the intake of 11 components: vegetables, fruit, fish, legumes or pulses, meat, whole grain products, low-fait dairy, olive oil, soft drinks, processed food and alcohol. Physical activity was assessed by the last item included in the Short Questionnaire to Assess Health-Enhancing Physical Activity (SQUASH; [52]), which refers to “average of days per week that the subject does physical exercise for at least 30 min”. Anthropometric measurements such us height and weight were taken to calculate the BMI (weight kilograms divided by height in square meters (Kg/m^2^). Blood pressure was also measured. Basal interviews included questions about the presence of somatic/chronic diseases (diabetes and stomach or intestinal ulcer were selected for the preset study), currently smoking, and alcohol consumption. Alcohol consumption was explored by using AUDIT test [53]. It consisted in a 10-item range from 0 to 4 to assess the risk alcohol consumption, dependence symptoms and harmful alcohol consumption. The sum of items ranging from 0 to 40 indicates the level of risk and dependence of alcohol consumption.

#### 2.4.5. Sociodemographic Variables

Country, age, sex, marital status and educational level were collected.

### 2.5. Outcomes and Data Analysis

Descriptive data were computed in terms of means, standard deviations, or percentages for the overall sample and per European Country. Differences between countries in sociodemographic, clinical, lifestyle and health as well as beverage consumption variables were examined. Beverages consumption was categorized as a dummy variable, and we defined it in three levels of frequency use. Soft drinks: <1 unit a week; 1 to 6 units a week; 1 or more units a day. Coffee and tea: <1 cup a day; 1 to 3 cups a day; >3 cups a day.

Binary logistic regression analysis was conducted with history of MDD as dependent variable and categorized as a dichotomous variable (history of MDD vs. no history of MDD). Odds ratios (ORs), 95% confidence intervals (CIs) and corresponding *p* values of lifetime depression presence associated with beverages consumption were calculated. Normality distribution of data was tested with Shapiro–Wilk test. Generalized linear models using a logistic regression formula were performed with all variables to explore the association between the frequency of beverages consumption as determinants entered jointly, and specific depressive symptom clusters (mood, cognitive and somatic) as outcomes measured by the IDS30-SR at 12-month follow-up. In addition, depression severity overall score was also explored as an outcome. R2, β coefficients, F value, 95% confidence intervals (CIs), and significance level (*p*) are reported. Baseline IDS30-SR score was included as covariate to interpret the regression coefficients as change compared with baseline. A stepwise backward formula-based model by AIC was performed to select final model. All analyses were adjusted for site, gender, sex, marital status, educational level (plus intervention group in longitudinal analysis), and in Model 1, and then, in Model 2, we added all identified potential confounders (BMI, MoodFood diet score, smoking, alcohol use, physical activity, high blood pressure, diabetes, and stomach or intestinal ulcer). We followed two strategies to evaluate the effect of the country variable. First, the country variable was introduced as a random effect, assuming that the observed variability could be conditioned by this variable, and second, the data were stratified by each country. Tests of assumptions of collinearity of the independent variables showed that multicollinearity was not a concern.

Categorical variables were coded as dummy or dichotomous variables: site (Germany, United Kingdom, Spain, The Netherlands); sex (male vs. female); educational level (lower: no studies or primary; middle: secondary studies; higher: university studies); intervention group (placebo; multi-nutrient supplements; placebo plus therapy; multi-nutrient supplements plus therapy), smoking (no vs. yes); high blood pressure (no vs. yes); diabetes (no vs. yes); stomach or intestinal ulcer (no vs. yes).

All statistical analyses were performed by IBM SPSS v24 (SPSS Inc., Chicago, IL, USA) and R version 3.6. The significance level was at a two-tailed probability <0.05.

## 3. Results

### 3.1. Descriptives

Table 1 describes the baseline data. Of the 941 participants, 275 were from Germany; 227 from United Kingdom; 215 from Spain; and 224 from The Netherlands. Mean age was 46.77 (SD = 12.94), ranging from 18 to 75; 75.3% were female; 48.1% of the sample had middle level of education, 42.2% higher education whereas the remaining 9.7% had a lower level. Lifetime prevalence of MDD was 33.2% (*n* = 312), with rates being significantly higher in the Netherlands and the United Kingdom sample in comparison with the other countries (*p* < 0.001). Higher alcohol use scores measured by AUDIT indicated a higher prevalence of MDD history diagnosis (*p* = 0.011). The severity of average depressive symptomatology was 21.74 (SD = 10.02) in the IDS30-SR, indicating mild depressive symptoms in the general sample. BMI average was 31.32 (SD = 3.95). A minority of the sample currently smoked (17.5%) and the mean of the score obtained in AUDIT test about alcohol use was 3.88 (SD = 3.67), indicating a normal consumption on average. On average, subjects did physical activity 3.61 (SD = 2.34) days/week. With regards to medical comorbidities, 30.8% of the sample suffered from high blood pressure, 5.2% from diabetes and 3.9% from stomach ulcer. Most of the sample (77.7%) drank less than 1 glass per week of carbonated/soft drinks with sugar, similar to the consumption of carbonated/soft drinks with NNSs (64%). In our sample, coffee is the most frequent drink; 56.7% of participants consumed 1 to 3 cups per day, whereas 66.6% of sample consume less than 1 cup of tea a day. 

The sample was heterogeneous between countries; chi-squared tests showed country differences in all the variables except for sex, diabetes and stomach or intestinal ulcer (see Supplementary Table S2).

### 3.2. Beverages Consumption and History of Depression

Table 2 presents the results of the consumption rates and its association with the presence of a past MDD diagnosis. Consumption of ≥1 unit a day of soft drinks with sugar was related to a higher prevalence of MDD history diagnosis (Odds Ratio (OR) = 2.28, 95% confidence interval (CI) = 1.17–4.36; R^2^_Tjur_ = 0.092) compared with <1 unit a week frequency consumption. The opposite association was found in the consumption of soft drinks with NNSs: ≥1 unit a day frequency of consumption was related to lower rates of MDD history diagnoses (OR = 0.55, 95% CI = 0.33–0.89; R^2^_Tjur_ = 0.092). These results are obtained after adjusting for site, age, sex, marital status, level of education (model 1). These results were also obtained in model 2 with the additional adjustment for BMI, MooDFOOD diet score, smoking, alcohol use, physical activity, high blood pressure, diabetes, and stomach or intestinal ulcer: OR for MDD history diagnosis among subjects who consumed ≥1 unit a day of soft drinks with sugar was 2.41 (95% CI = 1.20–4.77; R^2^_Tjur_ = 0.096) and OR = 0.55 (95% CI = 0.32–0.90; R^2^_Tjur_ = 0.096) for those who consumed ≥1 unit a day of soft drinks with NNSs. No associations were observed between frequent consumption of coffee and tea and a history of MDD diagnosis.

When we stratified these analyses per country, the associations between the different non-alcoholic beverages and MDD history diagnosis were only significant in Spain and The Netherlands. In the Spanish sample, consumption of ≥1 unit a day soft drinks with NNSs was associated with lower rates of MDD history diagnoses (OR = 0.20, 95% CI = 0.03–0.78; *p* = 0.042) in comparison with <1 unit a week frequency consumption. In The Netherlands sample, consumption of 1 to 3 cups of coffee a day, was related to higher rates of MDD history diagnoses (OR = 2.73, 95% CI 1.17–6.78; *p* = 0.024) compared with <1 cup a day consumption.

### 3.3. Beverages Consumption and Depression Severity per Symptoms Clusters

Generalized linear models showed no significant associations of the consumption of any studied beverages and overall depression severity score at 12-month follow-up. Mood, cognitive and somatic clusters entered separately to the analysis showed no significant effects associations with basal consumption rates in any of the non-alcoholic beverages studied (see Table 3). All analyses were adjusted for IDS30-SR baseline scores, site, age, gender, marital status, level of education, intervention group, with the additional adjustment of BMI, smoking, alcohol use, physical activity, high blood pressure, diabetes, and stomach or intestinal ulcer. Country variable was introduced as a random effect in the models. Results derived from generalized linear model analysis are shown in Table 3.

## 4. Discussion

The post-hoc analysis based on baseline data from the large-scale MooDFOOD prevention trial with the aim of exploring the relationship between certain non-alcoholic beverages (soft drinks sweetened with sugar or with NNSs, coffee and tea) and past MDD diagnosis showed that consuming at least one drink a day of soft drinks sweetened with sugar was associated with a higher prevalence of MDD history diagnosis than consuming less than one drink a day. In contrast, consuming at least one drink a day of soft drinks sweetened with NNSs was inversely associated with previous MDD diagnosis rates in comparison with lower consumption frequencies.

This is the first study to analyze the associations between a history of MDD diagnosis and the consumption of soft drinks, coffee and tea in overweight subjects with subsyndromal depressive symptoms. We found no association of coffee or tea consumption and previous MDD. Contrary to our findings, previous studies based on the fifth Korean National Health and Nutrition Examination Survey concluded that frequent consumption of coffee [37,38] and green tea ([37] was inversely associated with self-reported lifetime depression prevalence.

Regarding the second post-hoc analysis with the aim of exploring the potential differences in the severity of depressive symptom clusters at 12-month follow-up (mood symptoms, cognitive symptoms, and somatic/vegetative symptoms) in relation to the consumption of these non-alcoholic beverages at baseline, it showed no significant effects of the consumption of any studied beverage and depression severity neither on the global scale nor in mood, cognitive or somatic clusters.

This is the first study that has examined the association between non-alcoholic beverages intake and different specific depressive symptoms.

Results obtained about soft drinks consumption and their relationships with depression severity are partially consistent with those obtained in the literature. For example, [17] showed that a consumption of ≥4 cans/day of soft drinks, compared with non-drinkers, was associated (OR = 1.30; 95%CI: 1.17–1.44) with a higher prevalence of depression among older adults, both sweetened with sugar or with NNSs soft drinks. The observed inverse association between drinks consumption was supported by [27] where the pooled Risk Ratio (RR) for the high vs. low category consumption was 1.36 (95% CI 1.24–1.50).

Consistent with our results about coffee intake, a longitudinal study in an Italian population [54] found no significant association between coffee consumption and depressive severity. Contrary to these results, meta-analyses of observational studies [27,28,29] concluded that coffee consumption is significantly associated with a decreased risk of depression. As observed in the most recent meta-analysis [27] based on five cross-sectional and three prospective cohorts of observational studies, pooled RRs of depression diagnosis for the high vs. low categories of consumption were 0.73 (95% CI 0.59–0.90). For dose–response analysis, [28] report a nonlinear J-shaped relation establishing the peak of protective effect on depressive symptoms for 400 mL/day. In fact, a prospective study carried out in a Finish population [55] with a sample of more than 40,000 subjects, found that amongst the people who consumed more than 8 cups/day of coffee, the risk of suicide was 58% higher compared with moderate consumers (1–7 cups/day).

In the case of tea consumption, our results were consistent with findings obtained in the [28] meta-analysis and also in previous longitudinal [54] prospective [17] and cross-sectional [56] studies. In contrast, other meta-analyses of observational studies [27,28,34,35] concluded that frequent tea consumption was associated with a decreased risk of depression (e.g., RR = 0.71; 95% CI = 0.55–0.91; [27]. Regarding dose-response analysis, Dong et al. [35], found a linear association between tea consumption and the risk of depression, and, specifically an increment of three cups/day of tea consumption was associated with a decrease in the risk of depression of 37% (RR = 0.63; 95% CI = 0.55–0.71).

One potential explanation for the contrasting results is that we adjusted for diet quality by adding an overall diet score as a confounder to our model. Kawada et al. [57] reported the need to specify the effect of food intake over the lack of consistent results among studies. Another explanation is the low percentage of consumption of certain beverages in comparison with other studies. Specifically, more than the 70% of the sample consumed less than one soft drink sweetened with sugar a week, more than 60% less than one soft drink sweetened with NNSs and 64% consumed less than one cup of tea a day. These low percentages make it difficult to interpret the results. Higher consumption of NNSs in overweight and obese people has been reported by other authors (e.g., [58]). These authors found a positive significant association between NNSs consumption per day and BMI status. However, despite reducing sugar consumption, it was not associated with a lower caloric intake, which suggests a compensatory increase in energy intake from other food sources. Beverage consumption behavior in this population may be different compared to the general population, so we must be cautious with the findings of our study. Mechanisms underlying about the effect of fructose or sucrose from soft drinks and how it could be affecting depression remains unknown. Future biological research is needed to explain neurobiological or molecular concepts of our findings.

The present study has some limitations; first, the post-hoc design limits its applicability and generalizability and its cross-sectional design in the analysis of MDD history diagnosis limits the extraction of causal relationships of soft drinks, coffee and tea consumption with the risk of depression. Second, the low percentage of participants that consumed soft drinks frequently. Power calculations to determine the appropriate sample size would be useful to provide more confidence in the generalizability of these results. The third limitation is the non-differentiation of the sweetening method: what percentage or type of sugar and NNSs were included in the soft drinks; if coffee or tea were sweetened with sugar, sweetened with NNSs or non-sweetened. Several studies have linked sugar consumption with a risk of depression [18,19]. We have obtained different results in soft drinks dependent on if they are sweetened with sugar or with NNSs, and this may also possibly be similar in coffee and tea. Furthermore, it would be desirable to study the differences between caffeinated or non-decaffeinated coffee since caffeine properties are attributed with preventing depression along with other components of coffee. Finally, due to the specific characteristics of the sample, it is not a representative sample of the general population.

Our study has some strengths such as its multi-country sample. This is the first study that has analyzed associations between a history of MDD diagnosis and consumption of soft drinks, coffee and tea in subjects with subsyndromal depressive symptoms, focusing on several of the most consumed beverages, and analyzing soft drinks sweetened in different ways (sugar vs. NNSs). In addition, it is the first longitudinal study that has examined the association between beverage intake and differentiated specific depressive symptom clusters (mood, cognitive and somatic/vegetative symptoms). Finally, the inclusion of an overall nutritional index (MooDFOOD diet score) as a confounder was a strength.

These findings could add significant information in the growing body of literature on the role of healthy eating in the prevention of depression, helping health care providers to develop healthy eating recommendations and guidelines. It also provides greater emphasis for the importance of diet as a common modifiable risk factor in the prevention of depression in vulnerable population.

## 5. Conclusions

Soft drinks sweetened with sugar consumption at baseline were positively associated with MDD history diagnosis prevalence in a multi-country sample of overweight participants with subsyndromal depressive symptoms whereas soft drinks sweetened with NNSs were inversely associated for the high vs. low categories of intake. No relationships were found about coffee and tea consumption. Longitudinal analysis of mood, cognitive and somatic/vegetative clusters showed no significant effects associated with basal consumption rates in any of the non-alcoholic beverages studied. Further evidence is needed to address the effect the beverages on the MDD onset.

## Figures and Tables

**Table 1:** Baseline characteristics of the 941 included participants.

	Overall		History of Depression		
	(*n* = 941)	Range	Yes (*n* = 312)	No (*n* = 629)	*p* Value
Demographics					
Age, Mean, (SD)	46.8 (12.9)	18–75	46.5 (12.7)	46.9 (13.0)	0.634
Sex					0.800
Female, *n* (%)	709 (75.3%)		233 (74.7%)	476 (75.7%)	
Male, *n* (%)	232 (24.7%)		79 (25.3%)	153 (24.3%)	
Marital status: couple, *n* (% yes)	566 (60.1%)		199 (63.8%)	367 (58.3%)	0.125
Site:					<0.001
Germany, *n* (%)	275 (29.2%)		61 (19.6%)	214 (34.0%)	
United Kingdom, *n* (%)	227 (24.1%)		123 (34.9%)	104 (16.5%)	
Spain, *n* (%)	215 (22.8%)		56 (17.9%)	159 (25.3%)	
The Netherlands, *n* (%)	224 (23.8%)		72 (23.1%)	152 (24.2%)	
Level of education					0.115
Lower education, *n* (%)	91 (9.7%)		22 (7.05%)	69 (11.0%)	
Middle education, *n* (%)	453 (48.1%)		149 (47.8%)	304 (48.3%)	
Higher education *n* (%)	397 (42.2%)		141 (45.2%)	256 (40.7%)	
Lifestyle					
BMI, Mean (SD)	31.3 (3.95)	24–45	31.1 (3.74)	31.4 (4.05)	0.252
MoodFOOD diet score, Mean (SD)	51.7 (7.03)	24–71	51.9 (7.13)	51.5 (6.98%)	0.417
Smoking, *n* (% yes)	165 (17.5%)		56 (17.9%)	109 (17.3%)	0.885
Alcohol use, Mean (SD)	3.88 (3.67)	0–25	4.3 (3.94)	3.66 (3.51%)	0.011
Physical activity, Mean (SD)	3.61 (2.34)	0–7	3.73 (2.33)	3.55 (2.35)	0.259
Medical comorbidities					
High Blood pressure, *n* (% yes)	290 (30.8%)		83 (26.6%)	207 (32.9%)	0.058
Diabetes, *n* (% yes)	49 (5.2%)		14 (4.5%)	35 (5.6%)	0.586
Stomach or intestinal ulcer, *n* (% yes)	37 (3.9%)		8 (2.6%)	29 (4.6%)	0.179
Psychiatric characteristics					
Depressive Symptoms IDS30-SR, Mean (SD)	21.74 (10.02)	0–55	23.7 (10.1)	20.8 (9.83)	<0.001
Mood symptom cluster, Mean (SD)	7.49 (4.85)	0–26	8.46 (5.04)	7.00 (4.68)	<0.001
Cognitive symptom cluster, Mean (SD)	2.77 (1.99)	0–9	3.18 (2.01)	2.57 (1.94)	<0.001
Somatic symptom cluster, Mean (SD)	11.48 (4.88)	0–28	12.0 (4.74)	11.2 (4.93)	<0.001
Beverages consumption					
Soft drinks with sugar					0.317
<1/week, *n* (% yes)	731 (77.7%)		235 (75.3%)	496 (78.9%)	
1–6/week, *n* (% yes)	165 (17.5%)		58 (18.6%)	107 (17.0%)	
≥1/day, *n* (% yes)	45 (4.8%)		19 (6.1%)	26 (4.1%)	
Soft drinks with artificial sweetener					0.109
<1/week, *n* (% yes)	602 (64.0%)		199 (63.8%)	403 (64.2%)	
1–6/week, *n* (% yes)	232 (24.7%)		86 (27.6%)	146 (23.2%)	
≥1/day, *n* (% yes)	106 (11.3%)		27 (8.6%)	79 (12.6%)	
Coffee					0.956
<1/day, *n* (% yes)	290 (30.8%)		98 (34.4%)	192 (30.5%)	
1–3/day, *n* (% yes)	534 (56.7%)		175 (56.1%)	359 (57.1%)	
>3/day, *n* (% yes)	117 (12.4%)		39 (12.5%)	78 (12.4%)	
Tea					
<1/day, *n* (% yes)	627 (66.6%)		193 (61.9%)	434 (69.0%)	0.071
1–3/day, *n* (% yes)	244 (25.9%)		90 (28.8%)	154 (24.5%)	
> 3/day, *n* (% yes)	70 (7.44%)		29 (9.29%)	41 (6.52%)	

**Table 2:** Cross-sectional associations between basal beverage consumption frequency and history of major depression diagnosis.

Consumption Frequency	Model 1 ^a^		Model 2 ^b^	
	OR (95% CI)	*p* Value	OR (95% CI)	*p* Value
Carbonated/soft drinks with sugar (200 mL)				
<1/week	1.00 (reference)	-	1.00 (reference)	-
1–6/week	1.22 (0.80–1.84)	0.347	1.27 (0.82–1.94)	0.281
≥1/day	2.28 (1.17–4.36)	0.013	2.41 (1.20–4.77)	0.012
Carbonated/soft drinks NNSs (200 mL)				
<1/week	1.00 (reference)	-	1.00 (reference)	
1–6/week	1.10 (0.77–1.56)	0.612	1.10 (0.77–1.58)	0.594
≥1/day	0.55 (0.33–0.89)	0.019	0.55 (0.32–0.90)	0.020
Coffee (1 cup)				
<1/day	1.00 (reference)	-	1.00 (reference)	-
1–3/day	1.23 (0.88–1.72)	0.228	1.14 (0.81–1.61)	0.454
>3/day	1.08 (0.66–1.77)	0.750	1.00 (0.60–1.65)	0.994
Tea (1 cup)				
<1/day	1.00 (reference)	-	1.00 (reference)	-
1–3/day	1.17 (0.83–1.64)	0.374	1.18 (0.84–1.67)	0.341
>3/day	1.07 (0.61–1.85)	0.810	1.10 (0.63–1.92)	0.732

Abbreviations: OR, odds ratio; CI, confidence interval; NNSs, non-nutritive sweeteners; (a) adjusted for site, age, gender, marital status, level of education; (b) adjusted for site, age, gender, marital status, level of education, BMI, MooDFOOD diet score, smoking, alcohol use, physical activity, high blood pressure, diabetes, and stomach or intestinal ulcer.

**Table 3:** Generalized linear models on the associations between basal beverage frequency consumption and 12-month follow-up depression severity (total score, mood, cognitive and somatic clusters) measured by the IDS30-SR.

Consumption Frequency	Depression Severity (IDS-30) *		Mood Cluster *		Cognitive Cluster *		Somatic Cluster *	
	β (95% CI)	*p*	β (95% CI)	*p*	β (95% CI)	*p*	β (95% CI)	*p*
Carbonated/soft drinks with sugar (200 mL)								
<1/week	0.00 (Reference)		0.00 (Reference)		0.00 (Reference)		0.00 (Reference)	
1–6/week	−0.78 (−2.40–0.84)	0.342	−0.37 (−1.12–0.37)	0.325	−0.20 (−0.52–0.11)	0.202	−0.41 (−1.28–4.46)	0.350
≥1/day	−0.02 (−2.70–2.67)	0.989	0.34 (−0.89–1.57)	0.591	−0.25 (−0.77–0.27)	0.349	−0.65 (−2.09–0.79)	0.377
Carbonated/soft drinks with artificial sweetener (200 mL)								
<1/week	0.00 (Reference)		0.00 (Reference)		0.00 (Reference)		0.00 (Reference)	
1–6/week	0.40 (−0.97–1.77)	0.564	−0.06 (−0.69–0.57)	0.845	0.03 (−0.23–0.30)	0.797	0.26 (−0.47–1.00)	0.487
≥1/day	0.41 (−1.41–2.23)	0.659	0.05 (−0.78–0.88)	0.909	−0.07 (−0.42–0.29)	0.712	0.29 (−0.69–1.27)	0561
Coffee (1 cup)								
<1/day	0.00 (Reference)		0.00 (Reference)		0.00 (Reference)		0.00 (Reference)	
1–3/day	−0.46 (−1.73–0.81)	0.481	−0.04 (−0.62–0.54)	0.888	0.00 (−0.24–0.25)	0.993	−0.24 (−0.92–0.45)	0.498
>3/day	−0.18 (−2.05–1.70)	0.854	0.08 (−0.78–0.93)	0.862	0.02 (−0.34–0.38)	0.912	−0.15 (−1.15–0.86)	0.772
Tea (1 cup)								
<1/day	0.00 (Reference)		0.00 (Reference)		0.00 (Reference)		0.00 (Reference)	
1–3/day	−0.08 (−1.37–1.22)	0.908	0.02 (−0.58–0.61)	0.954	−0.06 (−0.31–0.19)	0.648	0.05 (−0.65–0.75)	0.887
>3/day	−0.06 (−2.19–2.07)	0.956	−0.32 (−1.29–0.66)	0.522	−0.04 (−0.45–0.37)	0.855	0.20 (−0.94–1.34)	0.729

* Adjusted for site, age, gender, marital status, level of education, BMI, MooDFOOD diet score, smoking, alcohol use, physical activity, high blood pressure, diabetes, and stomach or intestinal ulcer, intervention group and IDS30-SR basal score. CI, confidence interval.

## Supplementary Materials

The following are available online at https://www.mdpi.com/2072-6643/12/10/3202/s1, Table S1: IDS30-SR items included in the three symptom clusters. Table S2: Baseline characteristics of the 941 included participants per country.
