# Supplementary material for: Associations of Non-Alcoholic Beverages with Major Depressive Disorder History and Depressive Symptoms Clusters in a Sample of Overweight Adults
Source: Nutrients. 2020 Oct 20;12(10):3202. doi: 10.3390/nu12103202 (PMC7589496; doi:10.3390/nu12103202)
Supplement: Supplementary file 1 [file nutrients-12-03202-s001.pdf]

Table S1. IDS30-SR items included in the three symptom clusters.

Mood subscale	Cognitive subscale	Somatic subscale
Diminished capacity for pleasure or enjoyment	Concentration/decision-making problems	Aches and pains
Diminished interest in people/activities	Future pessimism	Appetite increase
Diminished quality of mood	Self-criticism and blame	Appetite decrease
Diminished reactivity of mood	Suicidal thoughts	Constipation/ diarrhea
Feeling anxious or tense		Diurnal variation with a worse mood in the morning
Feeling irritable		Early morning awakening
Feeling sad		Low energy level/fatigability
Interpersonal sensitivity		Other bodily symptoms
Leaden paralysis		Problems falling asleep
Panic/phobic symptoms		Problems sleeping during the night
		Psychomotor agitation
		Psychomotor retardation
		Reduced interest in sex
		Sleeping too much
		Weight increase
		Weight decrease

Table S2 Baseline characteristics of the 941 included participants per country.

	Germany (*n* = 275)	United Kingdom (*n* = 227)	Spain (*n* = 215)	The Netherlands (*n* = 224)	*p* value
Demographics					
Age, Mean, (SD)	43.47 (13.76)	50.45 (12.47)	43.34 (12.04)	50.38 (11.15)	<0.01
Sex, n (% female)	208 (75.6%)	181 (79.7%)	149 (69.3%)	171 (76.3%)	0.082
Level of education					
Lower education, n (%)	9 (3.3%)	9 (4.0%)	45 (20.9%)	28 (12.5%)	<0.01
Middle education, n (%)	185 (67.3%)	103 (45.4%)	90 (41.9%)	75 (33.5%)	<0.01
Higher education n (%)	81 (29.5%)	115 (50.7%)	80 (37.2%	121 (54.0%)	<0.01
Lifestyle					
BMI, Mean (SD)	31.13 (3.85)	30.64 (3.67)	32.20 (4.03)	31.38 (4.12)	< 0.01
Smoking, n (% yes)	63 (22.9%)	24 (10.6%)	44 (20.5%)	34 (15.2%)	< 0.01
Alcohol use, Mean (SD)	3.46 (3.00)	5.40 (4.64)	2.70 (2.48)	3.98 (3.73)	< 0.01
Physical activity, Mean (SD)	3.05 (2.21)	4.22 (2.11)	3.29 (2.57)	4.00 (2.30)	< 0.01
Medical comorbidities					
High Blood pressure, n (% yes)	92 (33.5%)	49 (21.6%)	65 (30.2%)	84 (37.5%)	< 0.01
Diabetes, n (% yes)	19 (6.9%)	11 (4.8%)	13 (6.0%)	6 (2.7%)	0.081
Stomach or intestinal ulcer, n (% yes)	10 (3.6%)	9 (4.0%)	9 (4.2%)	9 (4.0%)	0.991

Psychiatric characteristics					
History of depression, n (% yes)	61 (22.2%)	123 (54.2%)	56 (26.0%)	72 (32.1%)	< 0.01
Severity of depressive symptoms, Mean (SD)	20.18 (9.82)	22.11 (8.86)	26.20 (11.03)	18.99 (8.89)	< 0.01
Mood symptom cluster, Mean (SD)	6.67 (4.64)	7.51 (4.34)	9.73 (5.24)	6.31 (4.50)	< 0.01
Somatic symptom cluster, Mean (SD)	10.81 (5.01)	11.91 (4.49)	13.13 (5.17)	10.28 (4.31)	< 0.01
Cognitive symptom cluster, Mean (SD)	2.71 (1.89)	2.70 (1.84)	3.33 82.28)	2.40 (1.83)	< 0.01
Beverages consumption					
Carbonated/soft drinks with sugar					<0.01
<1/week, n (% yes)	195 (70.9%)	197 (86.8%)	158 (73.5%)	181 (80.8%)	
1-6/week, n (% yes)	61 (22.2%)	25 (11.0%)	44 (20.5%)	35 (15.6%)	
≥ 1/day, n (% yes)	19 (42.2%)	5 (11.1%)	13 (28.9%)	8 (17.8%)	
Carbonated/soft drinks with artificial sweetener					<0.01
<1/week, n (% yes)	207 (75.3%)	147 (64.8%)	113 (52.6%)	135 (60.5%)	
1-6/week, n (% yes)	51 (18.5%)	55 (24.2%)	76 (35.3%)	50 (22.4%)	
≥ 1/day, n (% yes)	17 (16%)	25 (23.6%)	26 (24.5%)	38 (35.8%)	
≥ 4/day, n (% yes)	7 (2.5%)	1 (0.4%)	5 (2.3%)	7 (3.1%)	
Coffee					<0.01
<1/week, n (% yes)	54 (19.6%)	58 (25.6%)	34 (15.8%)	43 (19.2%)	
1-6/week, n (% yes)	31 (11.3%)	32 (14.1%)	23 (10.7%)	15 (6.7%)	
1-3/day, n (% yes)	169 (61.5%)	113 (49.8%)	143 (66.5%)	109 (48.7%)	
≥ 4/day, n (% yes)	21 (7.6%)	24 (10.6%)	15 (7.0%)	57 (25.4%)	
Tea					<0.01
<1/week, n (% yes)	108 (39.3%)	96 (42.3%)	128 (59.5%)	50 (22.3%)	
1-6/week, n (% yes)	103 (37.5%)	30 (13.2%)	53 (24.7%)	59 (26.3%)	
1-3/day, n (% yes)	57 (20.7%)	66 (29.1%)	33 (15.3%)	88 (39.3%)	
≥ 4/day, n (% yes)	7 (2.5%)	35 (15.4%)	1 (0.5%)	27 (12.1%)	
